# Complete chloroplast genome sequences of *Acanthocalyx alba* and *Acanthocalyx nepalensis* subsp. *delavayi* (Caprifoliaceae)

**DOI:** 10.1080/23802359.2022.2124824

**Published:** 2022-09-23

**Authors:** Junjun Wang, Riza Zhao, Zhifeng Zhang

**Affiliations:** a School of Pharmacy, Institute of Qinghai-Tibetan Plateau, Southwest Minzu University, Chengdu, China; b Sichuan Provincial Qiang-Yi Medicinal Resources Protection and Utilization Technology Engineering Laboratory, Chengdu, China

**Keywords:** *Acanthocalyx alba*, *Acanthocalyx nepalensis* subsp. *delavayi*, chloroplast genomes

## Abstract

In this study, the chloroplast genomes of *Acanthocalyx alba* (Hand.-Mazz., 1925) and *Acanthocalyx nepalensis* subsp. *delavayi* (Franchet, 1885) were sequenced, and their total lengths were 148,720 bp and 149,253 bp, respectively. The *A. alba* genome contained two inverted repeat regions (IRs) of 21,849 bp, a large single-copy region (LSC) of 89,084 bp, and a small single-copy region (SSC) of 15,938 bp, whereas *A. nepalensis* subsp. *delavayi* contained two IRs of 21,736 bp, one LSC of 89,034 bp, and one SSC of 16,747 bp. The chloroplast genomes of both *A. alba* and *A. nepalensis* subsp. *delavayi* contained 109 genes, including 72 mRNA, 33 tRNA, and four rRNA genes. Phylogenetic analysis suggested that *A. alba* is in a clade with *A. nepalensis* subsp*. delavayi*. This study provides useful data for further phylogenetic studies of *A. alba* and *A. nepalensis* subsp. *delavayi*.

*Acanthocalyx* species plants are widely distributed in southwest and northwest China. Most of them are medicinal plants commonly used in Tibetan medicine (Zhang et al. 2011). *Acanthocalyx alba* (Hand.-Mazz.) M. J. Cannon (Cannon 1984) and *Acanthocalyx nepalensis* subsp. *delavayi* (Franchet) D. Y. Hong (Hong 2010) belong to the family Caprifoliaceae. They are the main sources of ‘Tibetan Acanthocalyx nepalensis’ in Tibetan medicines. Their aboveground parts have a good effect on symptoms such as joint pain, urinary incontinence, low back pain, vertigo, and mouth-eye deviation (Teng et al. 2002), and they are clinically used for the treatment of sore furuncle, purulent trauma, and tumors (Liu et al. 2006). As an important Chinese folk herbal medicine, *A. alba* has become increasingly endangered. However, most previous research has focused on its active ingredients. There are few reports about the population diversity and evolution of *A. alba* and *A. nepalensis* subsp. *delavayi* (Wu et al. 2014). Therefore, we sequenced their genomes and analyzed their genomic characteristics to provide a valuable reference for future studies on genetics research.

Two voucher specimens and DNA samples were deposited in the herbarium of Southwest Minzu University (contact person: Wang, email: 1299424380@qq.com). *A. alba* (voucher LY31245) was obtained from Luoge, Ganzi County, Ganzi Tibetan Autonomous Prefecture, Sichuan Province, China (N31°98′37.95″, E100°15′44.04″), and *A. nepalensis* subsp. *delavayi* (voucher LY31246) was obtained from Kangding, Sichuan Province, China (N30°03′13.99″, E101°24′01.27″). The collection and research of plant materials were carried out in accordance with national regulations. Total genomic DNA was extracted from fresh leaves using the CTAB (cetyltrimethylammonium bromide) method (Yang et al. 2014). A library was constructed and then sequenced with Illumina NovaSeq (Illumina Inc., San Diego, CA) (Jiang et al. 2011). De novo genome assembly was performed using the Spades software (Bankevich et al. 2012). Then, the assembled genome was annotated using the online annotation tool CPGAVAS2 (Shi et al. 2019). Finally, CodonW was used for codon preference analysis (Carbone et al. 2003).

The complete genome length of *A. alba* (OK323963) was 148,720 bp, including two inverted repeat regions (IRs, 21,849 bp), a large single-copy region (LSC, 89,084 bp), and a small single-copy region (SSC, 15,938 bp). The GC content of the whole genome was 38.22%, which was lower than the 43.70% GC content in the IR region, but higher than the 36.46% GC content in the LSC and the 32.98% GC content in the SSC. The complete genome length of *A. nepalensis* subsp. *delavayi* (OK323964) was 149,253 bp. The whole genome contained two IRs (21,736 bp), one LSC (89,034 bp), and one SSC (16,747 bp). The GC content of the whole genome was 38.25%, which was lower than the 43.82% GC content in the IR region, but higher than the 36.47% GC content in the LSC and the 33.22% GC content in the SSC.

There were 109 genes in the chloroplast genomes of *A. alba* and *A. nepalensis* subsp. *delavayi*, both consisting of 72 mRNA, 33 tRNA, and four rRNA genes. The sequence coding length for amino acids in the protein (CDS) of *A. alba* was 50,556 bp, and that of *A. nepalensis* subsp. *delavayi* was 50,562 bp. Their sequence coding lengths for rRNA and tRNA genes were 9046 bp and 2511 bp, respectively.

A phylogenetic tree was built to infer the phylogenetic position of *A. alba* and *A. nepalensis* subsp. *delavayi*, with *Diabelia* species as an outgroup (Figure 1). The chloroplast genomes of *A. alba* and *A. nepalensis* subsp. *delavayi* used in this paper were derived from sequencing, and 11 other chloroplast genomes were derived from the National Center for Biotechnology Information (NCBI) website. In this study, MEGA-X version 10.2.6 software was used to construct the phylogenetic tree using the maximum-likelihood method, with 1000 bootstrap replicates (Kumar et al. 2018). Compared with previous studies based on the complete plastomes of plants (Wang et al. 2020), we mainly sequenced the complete chloroplast genomes of *A. alba* and *A. nepalensis* subsp. *Delavayi* to investigate their phylogenetic relationships with other Caprifoliaceae plants, and focused on the relationship between *A. alba* and *A. nepalensis* subsp. *delavayi*. Analyses of the plastomes and chloroplast genomes were largely consistent, and *A. alba* and *A. nepalensis* subsp. *delavayi* clustered together with a bootstrap value of 100%. This research provides useful data for studying the phylogenetic relationships and genetic diversity of *A. alba* and *A. nepalensis* subsp. *delavayi*.

**Figure 1.**
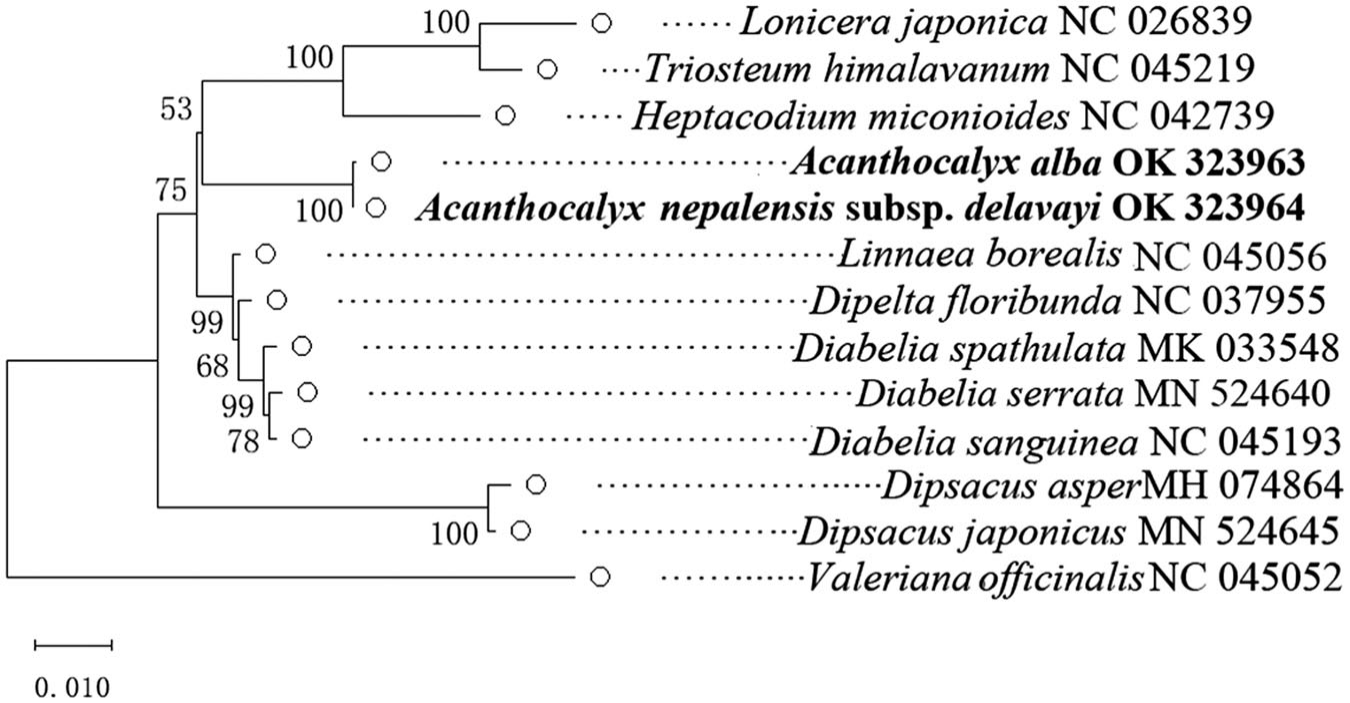
Maximum-likelihood tree of *Acanthocalyx alba*, *Acanthocalyx nepalensis* subsp*. delavayi*, and other Dipsacaceae species based on the whole chloroplast genome sequence.

## Author contributions

Junjun Wang performed the data analysis and wrote the manuscript; Riza Zhao contributed to the conception of the study; and Zhifeng Zhang made critical revisions to intellectual content. All authors agreed to be accountable for all aspects of the work.

## Geolocation information

Chengdu 610041, Sichuan Province, China.

## Data Availability

The genome sequence data that support the findings of this study are openly available in GenBank from NCBI at https://www.ncbi.nlm.nih.gov/ under the accession nos. OK323963 and OK323964. The associated BioProject, SRA, and Bio-Sample numbers are PRJNA765190-PRJNA765191, SRR17933251–SRR17938363, and SAMN21547079–SAMN21547080, respectively.
